# Cooperative Hybrid Modelling and Dimensionality Reduction for a Failure Monitoring Application in Industrial Systems

**DOI:** 10.3390/s25061952

**Published:** 2025-03-20

**Authors:** Morgane Suhas, Emmanuelle Abisset-Chavanne, Pierre-André Rey

**Affiliations:** 1 Univ. Bordeaux, CNRS, Bordeaux INP, I2M, UMR 5295, F-33400 Talence, France; emmanuelle.abisset-chavanne@ensam.eu (E.A.-C.); pierre-andre.rey@ensam.eu (P.-A.R.); 2 Arts et Metiers Institute of Technology, CNRS, Bordeaux INP, I2M, UMR 5295, F-33400 Talence, France; 3 AMVALOR, Arts et Métiers Institute of Technology, 151 Boulevard de l’Hôpital, 75013 Paris, France

**Keywords:** hybrid modelling, dimensionality reduction, classification, long short-term memory, failure monitoring, direct current motor

## Abstract

Failure monitoring of industrial systems is imperative in order to ensure their reliability and competitiveness. This paper presents an innovative hybrid modelling approach applied to DC electric motors, specifically the Kollmorgen AKM42 servomotor. The proposed Cooperative Hybrid Model for Classification (CHMC) combines physics-based and data-driven models to improve fault detection and extrapolation to new usage profiles. The integration of physical knowledge of the healthy behaviour of the motor into a recurrent neural network enhances the accuracy of bearing fault detection by identifying three health states: healthy, progressive fault and stabilised fault. Additionally, Singular Value Decomposition (SVD) is employed for the purposes of feature extraction and dimensionality reduction, thereby enhancing the model’s capacity to generalise with limited training data. The findings of this study demonstrate that a reduction in the input data of 90% preserves the essential information, with an analysis of the first harmonics revealing a narrow frequency range. This elucidates the reason why the first 20 components are sufficient to explain the data variability. The findings reveal that, for usage profiles analogous to the training data, both the CHMC and NHMC models demonstrate comparable performance without reduction. However, the CHMC model exhibits superior performance in detecting true negatives (90% vs. 89%) and differentiating between healthy and failure states. The NHMC model encounters greater difficulty in distinguishing failure states (83.92% vs. 86.56% for progressive failure). When exposed to new usage profiles with increased frequency and amplitude, the CHMC model adapts better, showing superior performance in detecting true positives and handling new data, highlighting its superior extrapolation capabilities. The integration of SVD further reduces input data complexity, and the CHMC model consistently outperforms the NHMC model in these reduced data scenarios, demonstrating the efficacy of combining physical models and dimensionality reduction in enhancing the model’s generalisation, fault detection, and adaptability. This approach has the advantage of reducing the need for retraining, which makes the CHMC model a cost-effective solution for motor fault classification in industrial settings. In conclusion, the CHMC model offers a generalisable method with significant advantages in fault detection, model adaptation, and predictive maintenance performance across varying usage profiles and on unseen operational scenarios.

## 1. Introduction

Monitoring failure in industrial systems is critical to ensuring their reliability and competitiveness. The task is to identify precursors of failure in the collected data, which can be used to build failure prediction models. Engineering can rely on physical models that represent the system itself, calibrated using appropriately collected data. However, many industrial models require continuous optimisation due to their inherent complexity involving non-linear relationships and numerous variables [1]. In addition, pure physics-based modelling can be costly to implement for complex systems. Furthermore, using systems under variable conditions requires constant updating of the implemented physical models. When used beyond the conditions of implementation, behavioural laws can quickly become obsolete.

In such cases, the relationships between the inputs and outputs of these complex systems can be modelled using industrial data, fundamental principles, or a combination of both, known as hybrid modelling. Hybrid modelling, which takes advantage of both principle-based and data-driven models, provides a balance between correct process generalisation and optimised computation based on historical factory data [2,3]. There are several architectures in the literature that combine these different models [4,5,6]. Hybridisation can be achieved by interacting between models, merging outputs from both types of models, and using outputs from one model to feed into the second [7].

**Figure 1:**
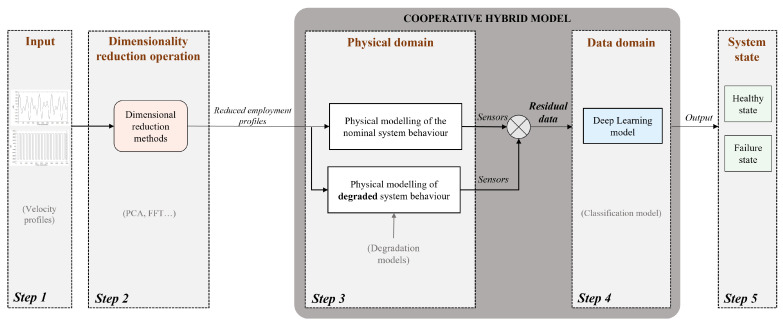
Generic framework for the implemented methodology.

This work builds on a literature review to define and complement several existing hybrid modelling approaches. In the first part of this paper, our first contribution is described: a new type of hybridisation between deep learning and physics-based models is proposed and called cooperative hybridisation (see Figure 1). Here, the physical knowledge model is used to simultaneously compare the nominal system behaviour with the actual behaviour derived from the system data. In this way, the physical knowledge of the nominal behaviour is integrated into the input data of the deep learning model. The deep learning model is trained solely on the difference between the behaviour of the system when it is in good condition and its behaviour when subjected to characterised disturbances.

To validate the method, it is applied to the task of fault diagnosis in DC electric motors and is subsequently referred to as the Cooperative Hybrid Model for Classification (CHMC). Nevertheless, it is a methodology that can be adapted to other tasks (prediction or classification). The study of the direct-current electric motor facilitated the implementation of the method, since it is extensively studied in the literature and its physical model representing its nominal behaviour is well known. The faults studied are bearing faults, which are the most common cause of failure [8]. The main advantage is the finer detection of bearing faults, resulting in a more pronounced drift of the signals recorded on the system.

The identification of faults in servomotor bearings is imperative, as such failures are among the most prevalent causes of servomotor dysfunction, resulting in diminished performance and augmented downtime. The present study concentrates on Kollmorgen’s AKM42 servomotor, as it represents a paradigmatic type of servomotor employed in industrial applications [9]. Furthermore, a test bench has been configured with this servomotor, which will facilitate the verification of the methodology on real data in future investigations.

However, the performance of hybrid modelling is highly dependent on the complexity of the hybrid architecture adopted and the extrapolation requirements specific to each application. In some cases, where hybrid models are not suitable for industrial processes, data-driven models may indeed offer better performance. However, incorporating knowledge of the physical process is crucial as it improves the transparency of machine learning algorithms [10]. Therefore, an additional step must be added to hybrid modelling to increase the generalisation ability of the model to new scenarios and raise the extrapolation limit of the model. It is common to observe performance gaps when transferring successful data-driven or hybrid models to other applications, especially when the characteristics of the input data set differ significantly from the one on which the model was trained [11]. This limitation is due to the inability of the model to generalise its predictions to new input data, highlighting the importance of developing methods to address this issue. The one considered hereafter is the dimensionality reduction of the input data [12], which is our second contribution of this work.

This approach involves isolating essential information from multiple collected data to improve system fault detection. Dimensionality reduction involves projecting data from a high-dimensional space into low-dimensional representations while preserving similarities between input data. Methods fall into two categories: feature selection and feature extraction. The former selects a subset of features without transforming the data, while the latter generates new features from the original data. These techniques are crucial for analysing complex data, as demonstrated by the use of various descriptive statistical methods. In particular, feature extraction captures non-linear relationships between variables, preserving information while reducing data size [13,14].

The motivation for this approach, developed in a second part, lies in the simplification of input databases to facilitate training and overcome difficulties in the generalisation of predictive models. The aim is to apply reduction methods to the input data of the hybrid model (usage profiles) in order to determine reduced bases that contribute to a satisfactory classification performance. In the case of industrial systems, in particular the direct-current electric motor, this is particularly useful when the same motor is used in more extreme conditions than those encountered in the sometimes costly modelling. Physical models then have great difficulty in adapting to real-world scenarios and produce inconsistencies.

Therefore, this approach, integrated into our hybrid modelling methodology, firstly increases the relevance of our analyses and predictions for use scenarios encountered during the learning phase. Secondly, by integrating these reduction methods, the CHMC model is able to satisfactorily detect bearing failures in new extrapolated scenarios. Given the shape of the usage profiles, which are periodic with a limited variety of occurrences, we will demonstrate that the most relevant method for this use case is the singular value decomposition (SVD) method. Although the methodology is applied to a specific industrial case, namely the electric motor, each step is precisely detailed to be reproducible on another system. It will then be necessary to adapt it to the physical knowledge of the system under study and to adjust the parameters of the reduction methods according to the available databases.

The main contribution of this work is based on the implementation of the CHMC that integrates real system data via physical models, allowing rapid model construction and adaptation to new usage profiles without loss of performance. The CHMC model, then combined with dimension reduction methods, enables the extrapolation of results to novel scenarios with a reduced number of input signals during the learning phase. The document is organised as follows: in Section 2, a review of the state-of-the-art with respect to the possibilities for the hybridisation of physical and data-driven models. Section 3 includes a complete description of the methodology, divided into two parts (hybridisation and dimensionality reduction methods), as well as the impact of the methodology on the fault diagnosis task. Finally, the conclusions of the work and the limitations of the method are discussed in a fourth section.

## 2. Related Works

### 2.1. Bearing Condition Monitoring Techniques

Condition monitoring techniques for rolling element bearings are divided into periodic (offline) monitoring and continuous (online) monitoring. The former measures vibration at specified intervals, while the latter performs continuous monitoring by comparing vibration levels with acceptable thresholds [15,16]. Due to its high cost, continuous monitoring is mainly used for critical applications. However, despite its reliability, continuous monitoring presents challenges such as data overload and the complexity of real-time analysis, requiring advanced signal processing techniques.

Various methods are used to detect bearing faults, including acoustic measurement, current and temperature monitoring, wear debris analysis, and vibration analysis. Acoustic emission detects structural changes due to cracking [17,18,19], but its sensitivity to external noise can lead to false alarms. Temperature monitoring detects failures due to bearing heating but may not detect early-stage failures. Motor current analysis detects variations in electrical noise associated with mechanical defects, providing a non-intrusive approach, but may lack precision in isolating fault locations. Wear debris analysis uses sensitive sensors to identify metallic particles in lubricants, making it effective at detecting severe faults, but it may not detect early-stage damage.

Vibration analysis, which is considered reliable, includes time-domain, frequency-domain and time-frequency-domain techniques [20,21,22]. Time-domain techniques use statistical parameters, which allow rapid fault detection, but lack visibility on the frequency distribution. Frequency-domain techniques are based on the passing frequency of each component of the sphere, and provide deeper insight into fault characteristics, but require expertise in signal interpretation. Time-frequency techniques, such as the Wigner–Ville distribution [23] or the wavelet transform [24], allow better analysis of non-stationary signals, although they require high computational resources.

Other approaches, including artificial neural networks (ANNs) [25] and fast Fourier transform (FFT), are commonly used in automation and vibration analysis [26,27]. ANNs provide robust fault classification but require extensive training data, whereas FFT is widely used for spectral analysis but can struggle with non-stationary signals. A critical evaluation of these techniques is essential to determine their suitability for specific applications, considering trade-offs between accuracy, computational cost and implementation feasibility.

### 2.2. Limitations of Fault Diagnosis Methods

In general, and according to the above methods, fault diagnosis literature presents two main approaches: those grounded in physical models and those based on data, depending on the level of prior process knowledge required [28]. Physical model-based approaches rely on a profound understanding of process physics (parameter estimation methods, parity relation methods, and fault tree methods). System data-based methods encompass expert knowledge, machine learning models, and statistical models.

However, both existing approaches, one primarily focused on physics and the other based on data manipulated by machine learning models, have their inherent limitations. Fault detection using a physical model yields precise detection when a precise physical model exists. Apparent difficulties then arise from the precision of the model itself, the impact of uncertainty on predictions, and the computational time required to solve complex mathematical models [29].

On the other hand, the data-driven framework is not entirely satisfactory as the quality of the response fundamentally depends on the quality of the data. Additionally, even if the data accurately represents the behaviour of the system, limitations lie in the interpretability of the solution, which is necessary for certifying models and extrapolating the domain of validity [30].

Given the limitations of both existing frameworks, hybrid modelling emerges as a promising approach that combines both types of methods. The new challenge lies in how to integrate physical knowledge into data-driven models. The different possible approaches are detailed in the subsections below before introducing the hybridisation proposed in this paper.

Thus, the model presented in this work will provide a solution to integrate the physical models of the system in order to both deal with the incomplete quality of the data representing the failures and improve the extrapolation capabilities of the model.

### 2.3. Hybrid Modelling Framework

**Figure 2:**
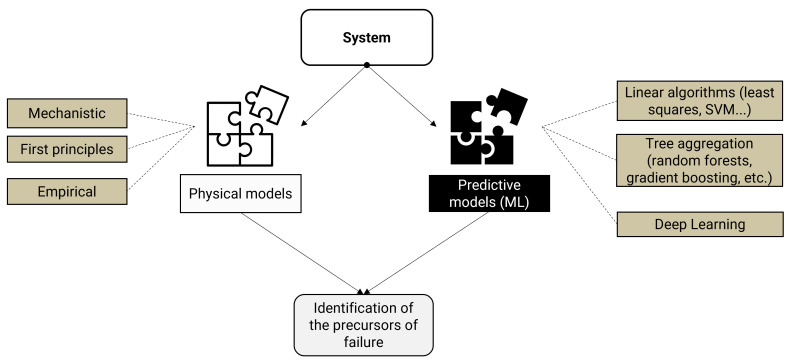
Principle of hybrid modelling.

Hybrid modelling consists of a combination of a theoretical model, which explains the system’s expected behaviour based on physical principles, and an artificial intelligence model, which adapts to the database (Figure 2). It is for this reason that they are also referred to as hybrid physics-based data-driven models (HPDM). The theoretical model, also known as the white box (WB), derives from knowledge of physical processes, and can be made up of first-principles or mechanistic models. Artificial intelligence models, on the other hand, are referred to as black boxes (BB), since they take no physical concepts into account in their construction. They model the behaviour of a system by finding correspondences between the inputs and outputs provided by the system. Although system modelling using WB alone requires little data, it is time-consuming and complex because some physical processes and/or parameters are not fully or only approximately known. WB models, on the other hand, provide full interpretability thanks to the analytical monitoring of physical quantities. In contrast, the implementation of BB models is generally less time-consuming, but requires a large amount of data and has a low degree of transparency and interpretability. As a result, hybrid modelling combines the advantages of both white-box and black-box modelling, according to Estrada Flores et al. [1]. For this reason, it is commonly referred to as a “grey box” (or hybrid model) [10].

The design of hybrid models depends on the modelling accuracy of the available physical models. The combination of white-box and black-box models can be implemented in series or in parallel, as described in depth in [31]. In a serial arrangement, the output of one sub-model is the input to the next sub-model. The hybrid serial model is generally used when not all the physical processes of the system are available due to their complexity, but a significant amount of data is still available [32]. Hybrid series models are mainly found in the fields of chemistry and energy, where WBs represent, for example, the conservation laws of reaction kinetics or capillary pressure laws. Several applications of these HMs are notably stated itn [31]. In Teixeira’s example [33], a BB/WB series structure is used to estimate one of the unknown reaction kinetics terms from experimental data. In the case of a WB/BB series structure, we can cite the example of [34] where the modelling of certain processes is incomplete due to undetected links between variables, but the WB model is sufficient to provide data to drive the BB model. This structure is also found in [35] where the WB model employed is the finite element method. On the other hand, in a parallel arrangement, the outputs of the WB and BB models are combined to produce the final output of the hybrid model. This arrangement is suitable when modelling via the WB models is insufficient to transcribe the system’s behaviour. The parallel combination of the two types of models therefore reduces the modelling error thanks to an estimation error existing in the WB model [36].

### 2.4. Hybridisation of Deep Learning Models

The term hybrid deep learning (HDL) was first introduced by Szegedy in [37] when he combined two deep learning (DL) models for an object detection problem. HDL models are indeed built by combining various artificial intelligence models to create a better performing DL model. Several performance criteria can be used to compare the models. The architecture of the hybrid model must be defined according to the problem at hand. The hybrid model is particularly useful when multiple inputs come from different measurements and are collected under several types (image, sequence, numerical value…) and several formats (continuous, discrete, 1D, 2D…). The hybrid model can then be composed of several neural networks that each manage a different type of input. Alternatively, a network can take care of extracting the features of the multiple inputs in a first step. Then, the concatenated features are passed to the next neural network for more targeted learning [38]. For example, if for a given problem, temporal dependencies are to be extracted from time series data, a recurrent neural network (RNN) should be considered. If, for this same problem, an analysis of spatial features could be beneficial then a convolutional neural network (CNN) can be grafted to the RNN [39]. An HDL model can also be useful for individual data sources. It is then used to exploit the specific potential of each type of DL model. In this case, the usual configuration is composed of two main steps: a first step for feature extraction and/or selection from the raw data and a second step for learning from the selected features. This is the case with the hybrid model presented in [40], which processes only images of faces to represent the calculation of similarity between two faces, taking into account the differences in lighting, age, etc. We also find this architecture in [41] which presents an HDL for real-time anomaly detection of an industrial system. As demonstrated in detail in [42], for the same data set, the hybridisation of DL models produces better performances compared to a model containing a single neural network.

### 2.5. Types of Hybrid Data-Driven Physics-Based Models

A categorisation of methods combining physics and data-driven models exists: (i) theory-driven methods; (ii) physics-informed methods; (iii) physics-augmented methods; (iv) physics-constrained methods; (v) simulation-assisted methods; and (vi) physics- guided methods.

A comprehensive review of these types of methods is given by Wang in [43]. For the sake of this work, we will just mention physics-informed methods, as this is the original HPDM method, and physics-augmented methods.

#### 2.5.1. Physics-Informed Neural Networks

The first category of hybrid data-driven physics-based model comprises physics-informed neural networks (PINN). PINNs are a promising approach in the field of machine learning. They combine concepts from physics and neural networks to solve complex problems. PINNs incorporate physical knowledge into their architecture to improve generalisation and reduce dependency on massive learning data. By using partial differential equations (PDEs) or conservation laws to constrain neural networks, PINNs are able to encode the underlying physical laws that govern a data set. For example, Wang et al. [44] use them to quantify natural convection flow fields from real data measurements. A usual method is to add constraints during the learning phase on an empirically constructed loss function. This approach has applications in many fields, such as fluid mechanics [45], thermodynamics, electromagnetism and particle physics. PINNs thus offer a significant potential to accelerate numerical simulation, optimize design processes or facilitate the discovery of new physical laws [46]. As supported by the pioneering framework PINNs of Raissi in [47], these methods are not seen as a replacement of classical numerical methods to solve partial differential equations (e.g., finite elements, Runge–Kutta methods, etc.). Rather, they provide insight into the construction of predictive algorithms to speed up the implementation. Although there is currently no clear formalisation for knowledge integration, a global taxonomy of informed neural network types is proposed by Kim et al. [48]. They are classified according to the type of neural network employed and how the physical knowledge is integrated into the network. In addition, a survey of the physical information provided and the physical problems solved in the literature based on PINNs is proposed in [49].

#### 2.5.2. Neural Network Augmented Physics Models

The second category of Hybrid data-driven physics-based presented comprises neural networked augmented physics models (NN-APM). NN-APMs are part of another approach that combines neural networks with physical models to also solve complex problems. Unlike PINNs, NN-APMs generally use existing and well-established physical models as a starting point. Neural networks are then used to improve or augment these physical models by capturing complex and nonlinear phenomena that are difficult to model analytically. NN-APMs can be used to perform predictions, simulations, or optimisation using both the information provided by the physical models and the training data. They are often used to combine physical models with experimental data or numerical simulations. In summary, NN-APMs improve existing physical models using neural networks, while PINNs incorporate physical knowledge into the design of neural networks themselves to solve physical problems. We are particularly interested in hybrid models for process modelling. Several applications, notably for chemical reactions, show that the hybrid model produces better prediction accuracy than without hybridisation [50,51].

Most approaches combining physical knowledge with data analysis techniques use hybrid architectures such as serial or parallel. Furthermore, the integration of physical knowledge is, in the first instance, carried out upstream of the data-driven model to estimate parameters or to generate data to be used as training data. In a second case, it is applied to the output data of the learning model to penalise the model’s loss function. In the final case, they are used to recalibrate the data-based model by estimating the prediction gap between the WB model and the BB model [52].

One contribution of our work is to propose a new architecture, described as cooperative, belonging to the NN-APM category. The term cooperative hybridisation refers to the simultaneous need for the physical behaviour of the system in nominal mode and data from the actual behaviour of the system, so that the data-based model can start the learning phase. As a result, physical knowledge is directly integrated into the input data of the data-driven model, leading to more accurate predictions.

## 3. Methodology

### 3.1. Overview

**Figure 3:**
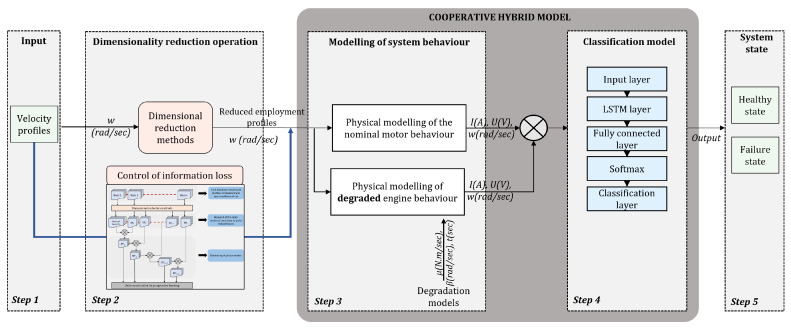
Schematic diagram of the overall methodology (CHMC model).

In this work, the development of the method presented consists of five steps as shown in Figure 3. Each step will be explained in this whole Section 3. The work represents a methodological approach to propose hybrid modelling of the direct-current (DC) electric motor system, and more specifically on the Kollmorgen AKM42 Servomotor, for diagnosing motor bearing faults [53]. The present study does not concentrate on the deterioration of a specific component; rather, it examines the distinction between nominal and faulty behavior. Regardless of the faulty component, bearing wear generates a resistive torque. To model this faulty behaviour, a resistive torque has been incorporated into the motor, which hinders the identification of the specific faulty component in the bearings.

It is referred to as a hybrid model since it relies on both physical models of the motor and real system data. In order to verify the results of the iteratively implemented methodology, the motor example was chosen because of its complete mastery of the underlying physical mechanisms and its widespread study in the literature. Because of the drawbacks, raised in Section 2.1, of each type of modelling when it is one-sided (physics-based only or data-based only), our contribution aims to partially address these issues. To this end, a new hybrid modelling architecture for defect classification is proposed. Indeed, after having modelled the motor using a multi-physics model, we will see how a predictive model based on the data is grafted to obtain the final hybrid model. In addition, in an attempt to counter difficulties in model extrapolation, an input data reduction process is nested before the hybrid modelling step (step 2 of Figure 3). Firstly, the methodology will therefore be tested on synthetic data. At each new step in the methodology, model performance will be evaluated. The steps will be described in the order in which they were built.

### 3.2. Proposed Hybrid Classification Model

After outlining the types of hybridisation found in the literature in Section 2, a new hybrid classification model structure is described below (step 3 of Figure 2). For the rest of this work, this hybrid model, based on data and physics, will be named as the Cooperative Hybrid Model of Classification (CHMC). Its special feature lies in the input data injected into the predictive classification model. To detect the presence of a fault in the electric motor, the classification model is trained during its learning phase on the discrepancy between the motor’s behaviour when in a healthy state and its behaviour when subjected to disturbances. A deviation that is too large will be diagnosed as a faulty motor state. On the other hand, a small deviation will result in a healthy diagnosis. The pre-processing of the input data injected into the hybrid model will be explained in detail below, after presenting the architecture of the classification model.

#### 3.2.1. Physics-Based Model Architecture

The first step of the modelling is to design a physics-based model representing the real system (step 3 of Figure 3). This subsection describes the modelling of the white box. In our case it represents the physical modelling of the DC motor as it is our application system, but it can be replaced by the physical modelling of any other known system. One has to note that the model introduced in the white box must be a model that is guaranteed to be accurate in order to transfer the modelling of the unknown in the black box. Like any motor, the DC motor is governed by physical equations encompassing the electrical, mechanical, and electromagnetic domains. Physical modelling is based on the operating equations presented below (see Equations (1)–(4)).

(1) $$\begin{array}{c} u\left(t\right)=e\left(t\right)+Ri\left(t\right)+L\frac{di(t)}{dt} \end{array}$$

(2) $$\begin{array}{c} e\left(t\right)=K_{e}w_{m}\left(t\right) \end{array}$$

(3) $$\begin{array}{c} J\frac{dw_{m}\left(t\right)}{dt}=C_{m}\left(t\right)-C_{r}\left(t\right)-fw_{m}\left(t\right) \end{array}$$

(4) $$\begin{array}{c} C_{m}\left(t\right)=K_{m}i\left(t\right) \end{array}$$

- u(t) : voltage applied to the motor terminals (V)
- e(t) : electromotive force (V)
- i(t) : current (A)
- $C_{m}\left(t\right)$ : motor torque (N·m)
- $C_{r}\left(t\right)$ : resistive torque (N·m)
- $w_{m}\left(t\right)$ : motor rotation speed (rad/s)
- *R* : motor armature resistance (ohm)
- *L* : motor armature inductance (H)
- *J* : motor inertia (kg·$m^{2}$)
- *k* : coefficient of viscous friction (N·m·$s^{-1}$)
- $K_{m}$ : motor torque constant (N·m/A)
- $K_{e}$ : electromotive force constant (V·s/rad)

The theoretical modelling of the electric motor is representative of its healthy behaviour. The healthy mode is used as a comparison to differentiate between nominal and faulty system behaviour. The physical models transcribed through the motor’s operating equations are modelled using MATLAB/Simulink software (version R2020b). Simulations are 500 s long, with a time step of 0.10 s. The aim of the physical model is to obtain operating data for our healthy system, with rotational speed as the control input. A rotation speed control loop $\left(w_{m}\right)$ was therefore implemented in the model to best represent the real system.

**Figure 4:**
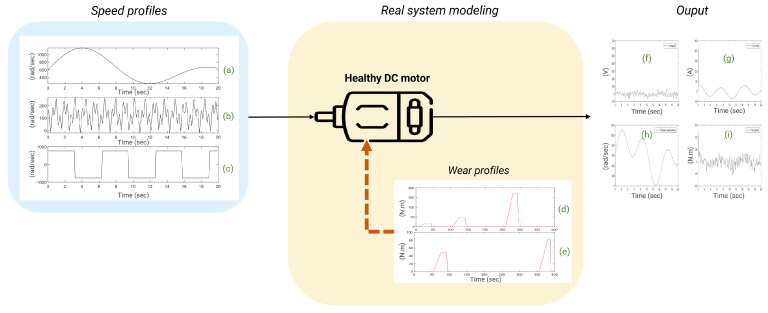
Physical modelling of the DC motor. (**a**) is a classical sinusoidal speed profile; (**b**) is an extrapolated sinusoidal speed profile; (**c**) is an impulsional speed profile; (**d**,**e**) represent examples of failure profiles; (**f**–**i**) represent the output data, i.e., the voltage, current, output speed, and torque, respectively.

In order to understand and control the impact of failures on motor behaviour, failures were introduced into the motor model via physical behaviour models found in the literature. The idea is therefore to degrade the multiphysics model resembling the healthy mode to obtain a degraded multiphysics model resembling the real behaviour of the system. Motor failures are physically modelled by resistive torques evolving over time [54]. Increasing resistive torque simulates bearing failure. In the course of a simulation, the motor can be subjected to up to 5 failure patterns, chosen at random for the sake of representativeness (Figure 4). With a view to fault prognosis in future work, this illustrates a failure profile that the motor may encounter during its service life. In this study, lifetime calculations are not included.

The input data for the physical motor models (healthy and degraded models) are time series representing motor speed profiles. The profiles are generated by varying the amplitudes and frequencies of various sinusoidal and pulsed signals. A table of 270 input profiles is constructed (Figure 4) with frequencies between 3 hertz and 40 hertz and magnitudes ranging from 400 rad/s to 550 rad/s. The output data of the physical motor models are multivariate time series which contain current, voltage, torque and speed.

The physical model output signals are amde noisy to model the measurement errors and random noise present in real systems. In order to verify the model’s responsiveness to noise, several levels of noise have been incorporated into the simulated data. These levels range from 10 dB to 20 dB, thereby providing signal-to-noise ratios (SNRs) that cover this range. The effectiveness of the model in reacting to noise can then be ascertained through this process.

#### 3.2.2. Data-Driven Model Architecture

This section is illustrated in step 4 of Figure 3 and corresponds to black-box modelling. In this work, LSTMs have been used because we are dealing with time series, but they can be replaced by other AI models depending on the use case.

**Figure 5:**
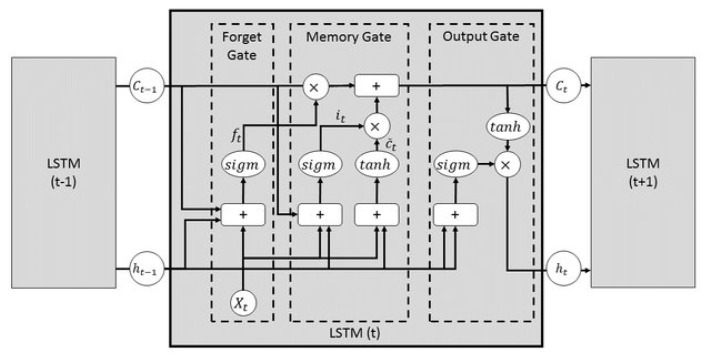
The structure of a single LSTM neuron [57].

Input data are pre-processed through a standardisation process, due to the different orders of magnitude of the predictor variables. After the pre-processing phase, the data passes through the first layer of the network: the long short-term memory layer (LSTM). The main role of an LSTM layer is to enable an RNN to capture long-term dependencies in data sequences using information regulation via control gate mechanisms and a memory cell. Unlike traditional RNNs, which can suffer from the problem of gradient disappearance over long sequences when back-propagating in time, LSTMs are designed to retain and use long-term historical information [55,56]. The LSTM cell comprises three major modules: the forget gate, the input gate (or memory gate), and the output gate (Figure 5). The forget gate decides what information is to be forgotten from the previous cell state. It considers the previous input state and the previous output. Next, the input gate determines what new information should be added to the cell state. It examines the previous input state and the previous output, then generates a candidate vector for the new information. Finally, the output gate defines the output of the LSTM layer according to the updated cell state. The output is then passed to the next network layer.

The input data to the neural network represent complete sequences of *n* predictor variables by time step. For example, at time step *m*, the input sequence *X* is of the form $X=\{x_{1}^{\left(m\right)},\ldots ,x_{n}^{\left(m\right)}\}$. The outputs are also complete sequences of all predictor variables that are predicted by time step. Knowing the complete behaviour of the signals over the observation period during the learning phase is crucial for predicting the evolution of motor failure. The RNN chosen for the model was therefore based on bidirectional long short term memory (BiLSTM). BiLSTM comprises two independent LSTMs with a similar fundamental framework. In BiLSTM, the learning sequence process contains forward and backward RNNs in which the connections between units form a directed loop and circulate data within the network so that previous information can be well preserved for future use [58].

**Figure 6:**
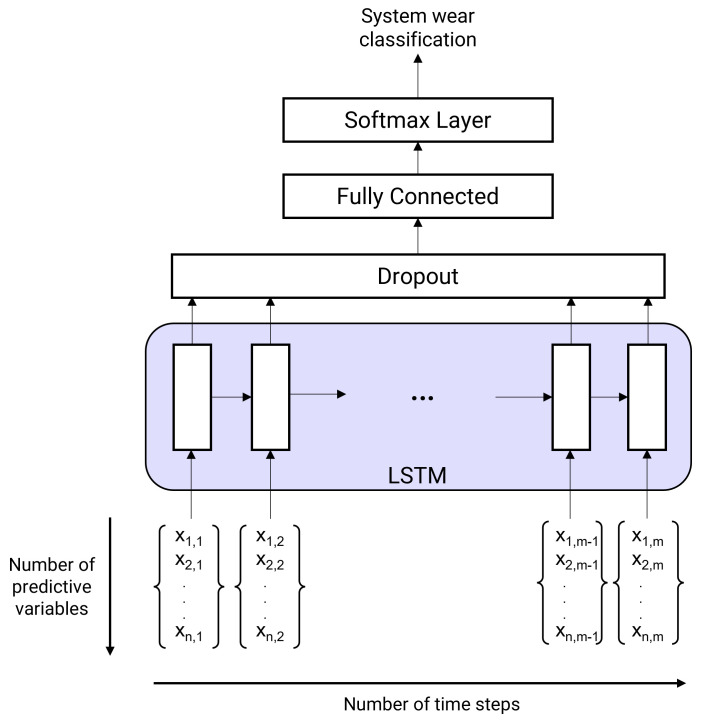
Schematic diagram of the hybrid classification model.

As shown on Figure 6, once the short and long-term dependencies have been learned, the data passes through the dropout layer, which is nested serially with the LSTM layer. This layer limits overfitting in deep learning models by randomly selecting neurons and disabling some of them during the learning process. The dropout layer is followed by a fully connected layer to produce the final output. In our multiclass problem, the final layer concerns the Softmax activation function, which transforms a real vector into a probability vector to associate the final label. The optimised parameters used in the experiments are listed in Table 1. The number of epochs has also been optimised to avoid overlearning. The network weights were adapted using the Adam algorithm, which is widely used in machine learning applications.

The model has been specifically trained to identify and distinguish the behavioural characteristics of a healthy physical model from those of a degraded model. In the event of disturbances being introduced into the system, these are dealt with by means of residual calculations. As the predictive model is better able to identify faults that appear progressively over time, isolated disturbances are not classified as such. In order to prevent such disturbances from being incorrectly classified as failures, the potential states of the system have been classified into three phases: ‘progressive failure’, ‘stabilised failure’ and ‘healthy state’. This ensures that only deteriorations that evolve over time are identified as failures.

The output of the classification model thus indicates the state of failure of the system under study, in particular that of a DC motor. The output includes labels that can represent the three distinct values listed above. A single fault type, a bearing fault, is generated with three different severity levels. For consistent classification, the model initially identifies a slight increase in data drift, which is described as ‘progressive failure’. If the signal drift no longer changes, although the system remains outside the healthy state, the failure is described as ‘stabilised’. If no drift is detected, the system is considered healthy.

#### 3.2.3. CHMC Model Architecture

A first work exposed in [53] consisted in classifying the complete sequence as faulty as soon as a faulty signal was detected within the sequence. Here, a finer-grained analysis is proposed, with classification by time step. The hybrid model identifies the motor failure condition based on the residuals between the output data of the degraded and healthy models. In this way, it locates a fault that has already occurred on the motor. The model therefore belongs to the NN-APM category.

Consequently, as the known healthy behaviour is incorporated into the white box, any uncertainty or inaccuracy in the model will be absorbed and modelled in the residual calculation with the real data. As shown in step 3 of Figure 3, we are interested in the residuals of the system data. In our case, the residuals result from subtracting the time signals of the modeled system subjected to faults (considered to be the real behaviour and ultimately represented by experimental data) and the time signals of the system in healthy behaviour (see Figure 4). It represents disturbances in the system, leading to potential failures. The training of the deep learning model is then based on the study of the difference between the data from the healthy system and the data from the faulty system. The hybridisation is therefore found in the interaction between the knowledge of the physical mechanisms that we have when the system is in nominal behaviour (step 3 in Figure 3) and the residuals that are analysed in fine detail (0.1 s steps) by a recurrent neural network (step 4 in Figure 3). The CHMC model created captures the short-term and long-term dependencies between the various residual data. It can be optimized if other sensors are added to the analysis later. The CHMC model thus obtained is able to identify the state of failure present in the system (classification) by analyzing the evolution of the residuals. It can also be used to predict future behaviour (predictive maintenance). The equations of the CHMC model are available below in (5) and (6).

The predictor data are an array of cells containing residual data sequences of the same length with four features. Each sequence consists of 5000 time steps. The predictor sequences are therefore matrices with four rows (one row for each feature) and 5000 columns (one column for each time step). The target data is a categorical vector of 5000 labels, corresponding to the three possible stages of motor failure at each time step: “healthy”, “progressive failure” and “stable failure”. An 80/20 distribution is applied for the learning phase and the test phase, respectively. This means keeping 864 sequences for learning and 216 sequences for testing the model. Among the 80% of sequences reserved for the learning phase, 10% are used for the validation phase.

Finally, hybrid modelling can be formalized using the following equations:

(5) $$\begin{array}{c} \Leftrightarrow \;\Theta_{sys}\left(\lambda \right)=\Theta_{\varphi }\left(\psi \right)+\varepsilon_{res}\left(\lambda \right) \end{array}$$

(6) $$\begin{array}{c} \Theta_{sys}\left(\lambda \right)=\Theta_{\varphi }\left(\lambda \right)+\Theta_{RNN}\left(\lambda \right) \end{array}$$

$\Theta_{sys}$ represents the system modelling depending on the usage profile λ. $\Theta_{varphi}$ is the physical modelling computed from the motor equations with healthy behaviour ψ. The ψ behaviour can, for example, be determined using several operating points or operating equations. $\varepsilon_{res}$ (Equation (6)), constituting the set of residuals, is the deviation between the nominal model and the real system. The final modelling of the system is obtained by exploiting the residuals as input data for the neural network $\Theta_{RNN}$ (Equation (6)).

#### 3.2.4. Results on Model Evaluation

To confirm the performance of the CHMC hybrid model, prediction results are compared on training and test data with a machine learning model built without including the physical knowledge of the electric motor, later called the Non-Hybrid Model of Classification (NHMC). The NHMC model is structurally analogous to the CHMC model, with the distinction that the former utilises simulated raw data as its input data, while the latter employs residual input data guided by physics and, more specifically, by nominal behaviour.

Due to imbalanced classes (15.23% of “progressive failure”, 11.98% of “stable failure” and 72.79% of “healthy state”), performance during the learning phase is evaluated according to the weighted cross-entropy personalized loss function and according to accuracy at the end of learning. As for the test phase, the two indicators calculated from the confusion matrix will be given in order to provide an overall view of the CHMC model’s performance. The first indicator calculated is accuracy, which gives the percentage of correct predictions over the total number of predictions. The second one is the $F_{1}-Score$ metric which is more representative for the imbalanced classes problems than the accuracy. In fact, the accuracy does not take into account the true negatives. Since this is a multi-class problem, the final $F_{1}-Score$ was obtained by averaging the $F_{1}-Score$ of each class c (see Equation (7)) .

∀c∈[1,3],

(7) $$\begin{array}{c} F_{1}-Score_{c}=2.\frac{precision_{c}*recall_{c}}{precision_{c}+recall_{c}} \end{array}$$

**Figure 7:**
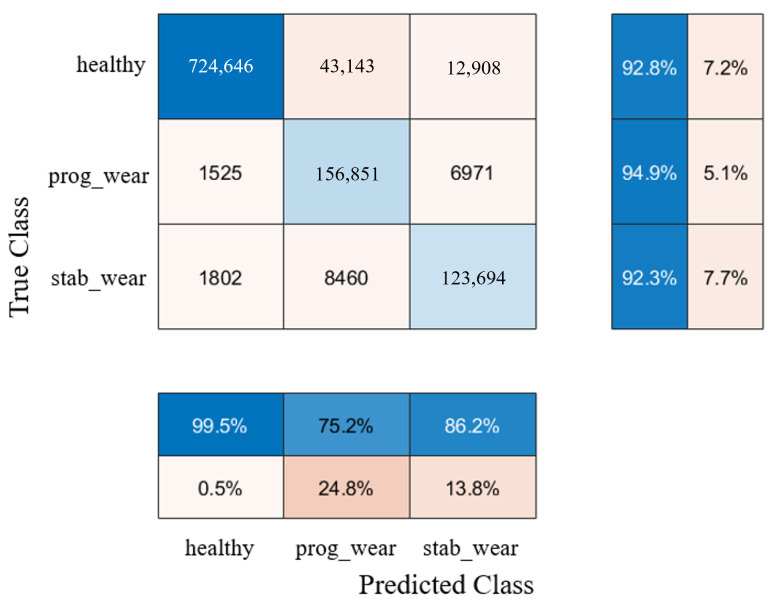
Confusion matrix of the NHMC.

**Figure 8:**
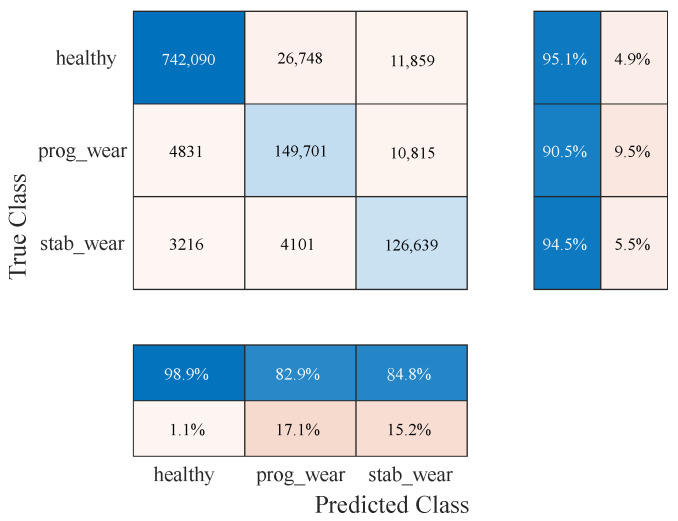
Confusion matrix of the CHMC.

With regard to classification on usage profiles similar to those encountered during the learning phase, the performance of the CHMC model is equivalent to that of the NHMC model, although the CHMC model performs slightly better in detecting true negatives (see Figure 7 and Figure 8). Knowledge of the motor’s nominal physical behaviour enabled to distinguish the true negatives for each class more finely (see $F_{1}-Score$ in Table 2).

The NHMC model has more difficulty differentiating the onset of failure from a healthy state (see Table 3). For future predictive maintenance, where the aim is to avoid unnecessary failure warnings, a CHMC-type model will therefore be preferred. These confusion matrix results (Figure 7 and Figure 8) show first of all that integrating physical models into system modelling is beneficial. In addition, the CHMC model can better support the BB model’s decision-making in terms of better understanding the observed deviation in behaviour, without impacting classification performance. The physical models effectively serve to make the model more explicable, by identifying which sensor may have contributed to the system state decision, thanks to a higher difference in behaviour. Having demonstrated the benefits of this type of hybrid modelling, and with a view to extrapolating input data, we now need to test the performance of the CHMC model when motor usage profiles are far removed from those encountered during the learning phase.

The model’s performance was subjected to a series of tests designed to assess its functionality under conditions of varying noise levels. To this end, the training data was maintained constant, whilst the test data was simulated by applying different SNRs. The $F_{1}-Score$ presented in Table 4 is the global $F_{1}-Score$, i.e., it is weighted according to the number of samples per class.

To implement hybridisation in another system, each of the substeps in Section 3.2 must be repeated. Firstly, the physical model is replaced by the physical knowledge of the new system so that the healthy behaviour (known as the nominal behaviour) can be identified and validated. Secondly, labelled real data are required to start the learning phase. These data must be collected on components that show the onset of failure. It is preferable that the failure is not too advanced such that the model can more accurately detect drift as it is trained. The learning parameters should also be optimised according to the application.

### 3.3. Dimensionality Reduction Operation

Dimensionality reduction methods are useful for visualising and processing high-dimensional data sets, while retaining as much variance as possible in the data set [13,14]. Indeed, the objective is to find a reduced basis of the data that explains all the variability of the input data. The relevance of this basis for failure prediction will be verified a posteriori. There are two main categories for dimensionality reduction which are feature selection and feature extraction [59]. Feature selection consists of selecting a subset of features by performing no data transformation, while feature extraction creates a new set of features from the input data.

In this work, some of the most popular techniques used in descriptive statistics are applied, but other techniques could have been used such as those cited by Sorzano [60]. These methods, belonging to the feature extraction category, allows the capture of non-linear relationships between variables, and many of them are invariant to monotonic transformations of the input variables [61]. Feature extraction methods have been selected in order to reduce the size of the input data while losing as little information as possible. Indeed, selection methods could also have been applied, but they require more knowledge of the data beforehand. Furthermore, our methodology provides for the integration of knowledge during the physical modelling of the system and not on the input data of the physical model. This section describes step 2 in Figure 3. The methods studied will be applied to the table of 270 speed profiles mentioned above. The information loss control loop during the transition to a reduced input profile base will not be dealt with in detail here. It is included in step 2 of Figure 3 and will be used to verify in future work that the reduced bases are appropriate for representing most usage profiles of the industrial system. If this is not the case, it will provide a warning that the extrapolation error is too great. The performance of subsequent steps will then no longer be guaranteed by the proposed hybrid model.

The application of the reduction methods to the input data of the physical model can be generalised as follows. In order to retain the essential information in the usage profiles, a certain number of modes must be selected for each method. To do this, we define a percentage of variability to be retained in the profile database. The eigenmodes are ranked by importance and the most important are retained to respect the variability threshold. The signals are then reconstructed according to the most important modes.

#### 3.3.1. Filtering Methods

First, linear filtering techniques are applied to study the importance of the frequency ranges contained in the velocity profiles injected into the physical model of the system. The method applied consists of filtering the input signals with a low-pass filter in the first instance. The high frequencies, i.e., the frequencies above the chosen cut-off frequency, were removed to leave only the low frequencies. The cut-off frequency was chosen after studying the distribution of the input signal spectra. It was selected as the average frequency of the spectra.

The low-frequency signals are reconstructed in the time domain using the inverse fast Fourier transform and fed into the hybrid model as input data. The filtered signal reconstructed from the frequency domain is equivalent to the filtered signal in the time domain. Similarly, the input signals are filtered in a second step using a high-pass filter and then also reconstructed in the time domain [62].

#### 3.3.2. Fast Fourier Transform

To reduce the initial base of the input signals, the frequency content of the signals can be compared. The time series are transformed into the frequency domain according to their spectrum via the discrete Fourier transform (DFT), defined as below [63]. Let us take a signal x[n] containing *N* samples. Its DFT is then defined by the following:

(8) $$X\left[k\right]=\sum_{n=0}^{N-1}x\left[n\right]\exp^{\left(-j2\pi \frac{kn}{N}\right)},0\leq k\leq N-1$$

The DFT is applied to all input signals. The peaks, corresponding to the harmonics, are identified on each spectrum of each signal and their respective frequencies are recovered. To identify the peaks, a minimum amplitude threshold between the highlighted peak and its neighbours is defined. The threshold was chosen to retain only the most important peaks for characterising the spectrum without suppressing any information. A limit of the first 15 harmonics is set for the spectrum analysis. Redundant spectra are removed to keep only those with unique frequency content. A total of 240 unique spectra containing several peaks are retained. Their index is recovered, and the unique signals are isolated from the complete base to form the reduced base composed of the spectra.

#### 3.3.3. Singular Value Decomposition/Principal Component Analysis

Another method commonly used in dimensional reduction is the singular value decomposition (SVD) method [64,65,66,67]. It is a non-parametric technique which allows the expression of a matrix $A\in R^{m*n}$ using two orthogonal matrices $U=\left[u_{1},u_{2},\ldots ,u_{m}\right]\in R^{m*m}$ and $V=\left[v_{1},v_{2},\ldots ,v_{m}\right]\in R^{n*n}$ such as the following:

(9) $$A=U\sum V^{T}$$

where $\sum =[diag\left(\theta_{1},\theta_{2},\ldots ,\theta_{q}\right),O]$, *O* is a zero matrix and q=min(m,n). The parameters $\theta_{i}\left(i=1,2,\ldots ,q\right)$ are the singular values of A and $\theta_{1}\geq \theta_{2}\geq \ldots \geq \theta_{q}>0)$.

**Figure 9:**
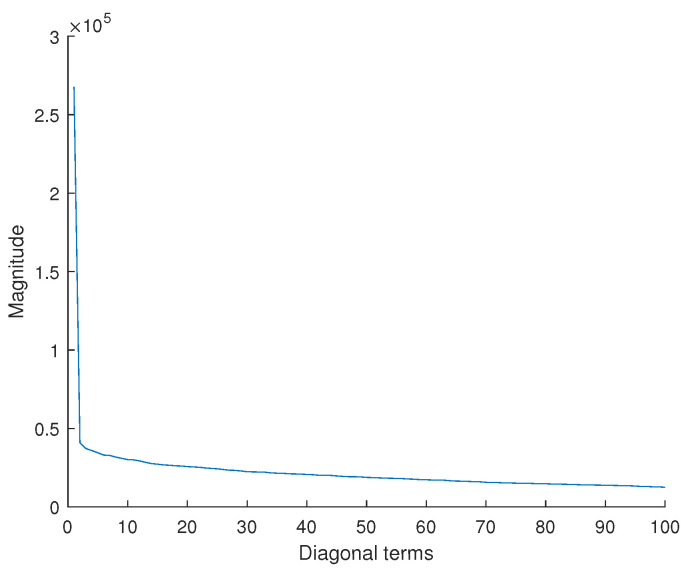
Mode energy of the input signals.

SVD was applied on the basis of the initial input data, but principal component analysis (PCA) could also have been used. Indeed, the two techniques are closely related. PCA works by finding the eigenvectors of the covariance matrix and ranking them by their respective eigenvalues. The latter are the squares of the singular values found by the SVD [63,68]. To estimate the number of input signals to be retained for training the hybrid model, the SVD method is applied to the set of signals (Algorithm 1). The diagonal values of the S-matrix obtained constitute the spectrum of singular values. The magnitude of a singular value reflects its importance in explaining the data. According to Figure 9, the first 20 modes of the signals are the most important to explain the data without losing information in the frequency content. In the following, we have limited ourselves to the first 10 modes. The 20 input signals that best explain the variability of the data are then reconstructed in the time domain [69]. After describing and testing each method, the dimensional reduction methods are combined.
**Algorithm 1** Methodology for reducing the usage profile database (SVD example)  **Inputs:** $Basis_{speed\_profiles},num_{modes}$ $U,S,V\leftarrow SVD(Basis_{speed\_profiles})$ $S_{reduc}\leftarrow S$ $U_{reduc}\leftarrow U$ **for** $i=1:length(S_{reduc})$ **do**     **if** $i>num_{modes}$ **then**         $S_{reduc}\leftarrow 0$         $U_{reduc}\leftarrow 0$     **end if** **end for**

#### 3.3.4. Study of the Most Influential Spectra Using PCA

**Figure 10:**
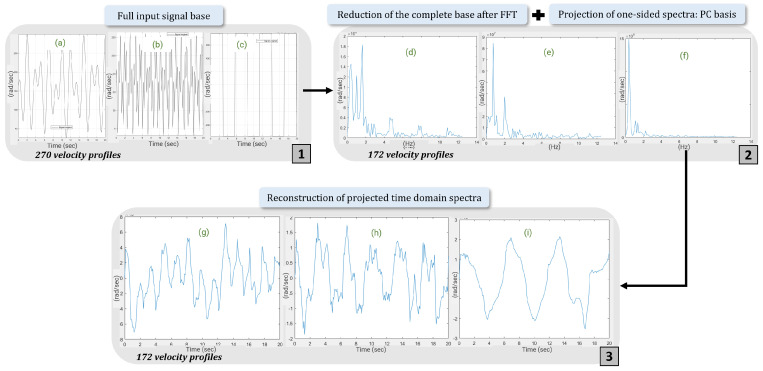
PCA reconstruction of input signals. (**a**–**c**) represent input speed profiles; (**d**–**f**) represent the frequency spectra of the speed profiles (**a**–**c**), respectively; (**g**–**i**) represent the spectra (**d**–**f**), respectively, after projection onto the principal component basis.

After describing and testing each of the methods, the dimensional reduction methods are combined. Once the DFT has transformed the input signals into spectra, the next step is to determine which are the most influential. For this purpose, the PCA method is applied to these spectra. The most influential spectra are then analysed using PCA. It highlights families of spectra by exploring linear relationships across all spectra. This amounts to solving a problem of maximizing the projection variance of the data. Figure 10 shows that after applying PCA to the spectra obtained via DFT, the results obtained previously with SVD are repeated, i.e., the first 20 components are sufficient to explain more than 95% of the variability of the 270 usage profiles. We conclude that by reducing the number of input signals forming the training base of the predictive model by 90%, the essential information contained in the initial data is preserved. Subsequently, an analysis of the frequencies of the first harmonics of each spectrum confirmed a narrow range of frequency distributions. This may explain why the first 20 components are sufficient to explain the variability in the data.

### 3.4. Impact and Results of Reduction Techniques

#### 3.4.1. Evaluation Process

The CHMC model demonstrated equivalent performance for a multi-class classification task compared with a non-hybrid model. These results are more valid for motor usage profiles like those in the training data. Now, the challenge of this section is to show the adaptation capabilities when new usage profiles are applied to the motor. More specifically, new signals, serving as input data for the physical model and for the degraded physical model, are created so that they differ from the initial base of usage profiles described in Section 3.2.1. To this end, the study will focus on the performance of the reduction techniques obtained through the detection of artificial faults by the classification model. To ensure that the reduced bases obtained by the reduction methods are relevant, i.e., that it synthesizes the essential information contained in the usage profiles, the model must achieve the same classification performance with the reduced bases of the input data. After projecting the new signals onto the reduced bases obtained in Section 3.3.4, the model is not trained again with these new signals. The weights of the CHMC and NHMC models are those memorized during training in the Section 3.2.4. In fact, the models have been trained with the reduced basis composed of reconstructed initial usage profiles. The trained models are then used to classify the new projected signals.

First, the extrapolation capacity of the CHMC model is measured by evaluating the classification of new usage profiles without projection onto the reduced bases. It is then compared with that of the NHMC model. The initial base used for training was composed solely of sinusoidal signals. To test the maximum extrapolation limits of the model, the new input signals contain pulse waves as well as sinusoidal signals with distinct amplitudes and frequencies. To encompass more usage profiles, the frequency and amplitude ranges are increased so that the ranges of the initial profiles are included in the new ranges. The frequency range has been increased by 42% compared with the initial base. On the other hand, the amplitude range has been increased by only 2.5%, as it is limited by the operating range of the motor studied.

**Figure 11:**
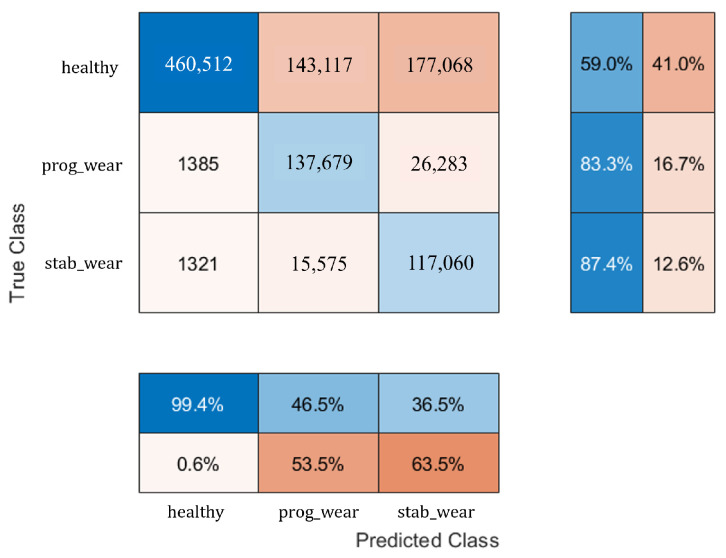
Classification of the CHMC of the extended profiles/before projection model.

**Figure 12:**
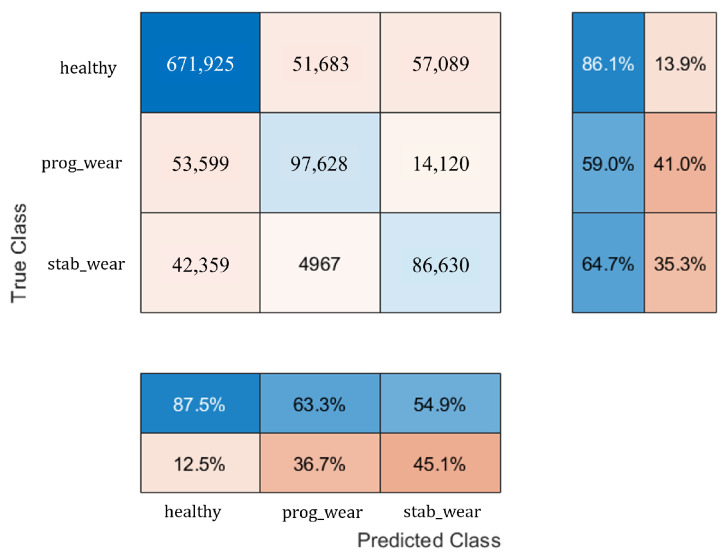
Classification of the NHMC of the extended profiles/before projection model.

Figure 11 shows that the CHMC model tends to classify true positives well but has greater difficulty in detecting true negatives. The NHMC model, on the other hand, is slightly better at detecting true positives but more prone to diagnostic errors (Figure 12). As a result, the CHMC model performs better when faced with new usage profiles.

**Figure 13:**
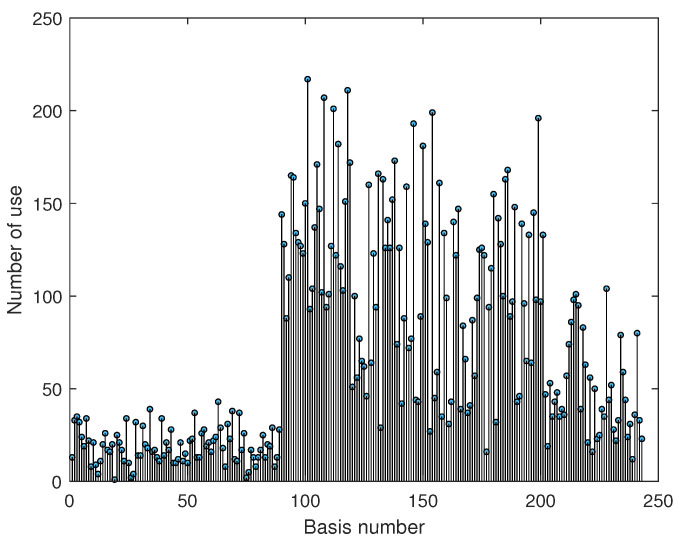
Contribution of basis to reconstructed signals.

In the second step, a more thorough verification is specified by expressing these new input signals in terms of the databases presented in the section above and proving that the model manages to adapt quickly. These new usage profiles are produced by modifying the frequencies, amplitudes and types of signals compared with the signals in the initial database. Figure 13 highlights the bases that contributed most to the reconstruction of the new signals after projection. This involves identifying the 60 most important coefficients for each new input signal during projection onto the initial base. The 60 corresponding bases are then isolated, and each of their number of appearances are counted.

The procedure, identical for each reduction method, has been automated as specified in Algorithm 2. To illustrate this procedure, let us take the example of the singular value decomposition (SVD). To express the new data in the reduced databases ($B_{reduc}$), an orthonormal basis ($Basis_{ON}$) is constructed for each method presented above. A column space of A (data matrix after reduction via a method) is constructed using the 30 columns of U corresponding to the most influential non-zero singular values for the initial data set (of the 270 motor usage profiles). The new B data ($B_{new}$) are then projected into this orthonormal base to form the C matrix. C is a representation of matrix B in the column space of U. To understand what C means in the initial space ($C_{tempo}$), i.e., time space in this case, we multiply the columns of C by the orthonormal basis [70]. These data are then fed into the hybrid motor fault classification model (CHMC). Similar reasoning is applied to the other reduction methods.
**Algorithm 2** Algorithm to study the impact of reduced basis on classification  **Inputs:** $Basis_{full},options_{in},Basis_{ON},B_{new}$ $Basis_{full_{mix}}=shuffle\left(Basis_{full}\right)$ $B_{reduc}=method_{reduc}\left(Basis_{full_{mix}}\right)$ $residuals=CHMC_{model}\left(B_{reduc}\right)$ $\left[lstm_{NN},param_{out}\right]=train\left(residuals,options_{in}\right)$ $P_{Basis_{ON}}\left(B_{new}\right)=transpose\left(Basis_{ON}\right)*B_{new}$ $C_{tempo}=Basis_{ON}*P_{Basis_{ON}}\left(B_{new}\right)$ $\left[residuals_{new},y_{test}\right]=CHMC_{model}\left(C_{tempo}\right)$ $y_{pred}=classify\left(residuals_{new},lstm_{NN}\right)$ Computation of : $Matrix_{Conf}\left(y_{pred},y_{test}\right)$

#### 3.4.2. Models Evaluation

After projection of the new data onto the databases obtained with the different reduction methods, the CHMC model delivers a classification performance that is stable from one projection to the next. More precisely, almost identical performances are obtained despite different base sizes. On the other hand, the NHMC model presents difficulties of generalisation when used on extrapolated input data. This is particularly true when projecting data based on the 20 selected spectra or on the basis of the 20 modes constructed by the SVD method.

For each reduction method applied to the new profiles, the models will be compared according to the accuracy and $F_{1}-Score$ criteria. However, as explained in Section 3.2.4, the $F_{1}-Score$ metric turns out to be the most relevant and representative for discerning the models. To continue with the example of reduction using the SVD method, the classification of these projected data onto the basis of the CHMC model produces an $F_{1}-Score$ of 91.93%, compared with 72.60% for the NHMC model. In the case of DFT, a first study of the initial database containing elementary usage profiles enabled us to build a database of 172 spectra, after eliminating duplicates. While maintaining equivalent performance, the SVD method enables the extraction of just 20 unique modes. To achieve such a reduction by focusing the study of spectra, the PCA method was applied in the section above to identify the 20 most influential spectra. After projection of the new profiles onto this basis and classification by the two models, this is the least effective reduction method. The 20 principal components constructed from the spectra are incomplete for generalizing information to new data. This method provides less conclusive results than without working on a reduced basis.

**Figure A1:**
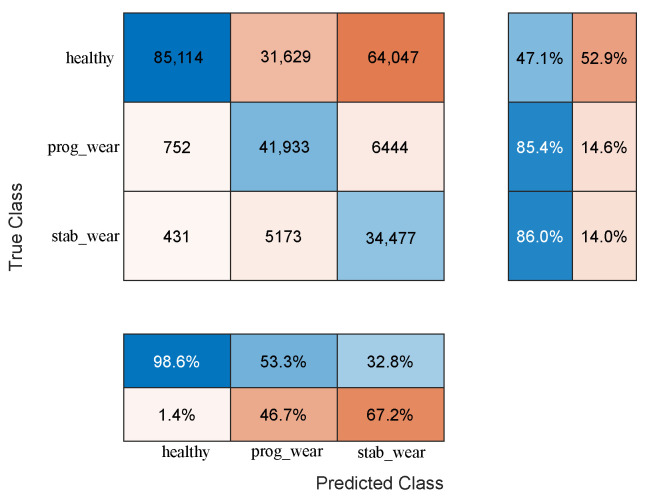
Low-pass filtering—NHMC model.

**Figure A2:**
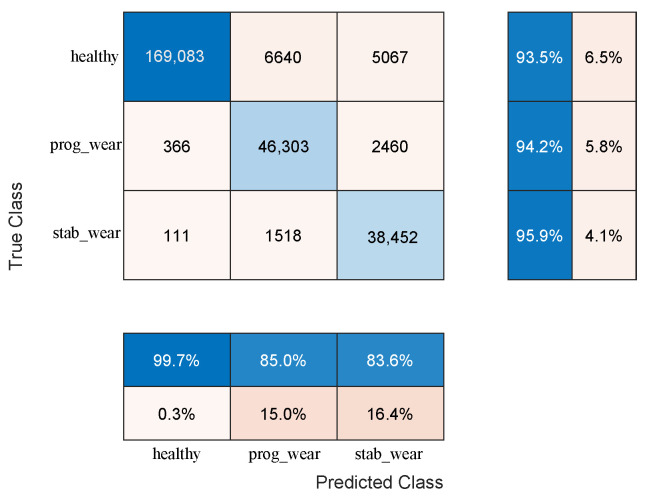
Low-pass filtering—CHMC model.

**Figure A3:**
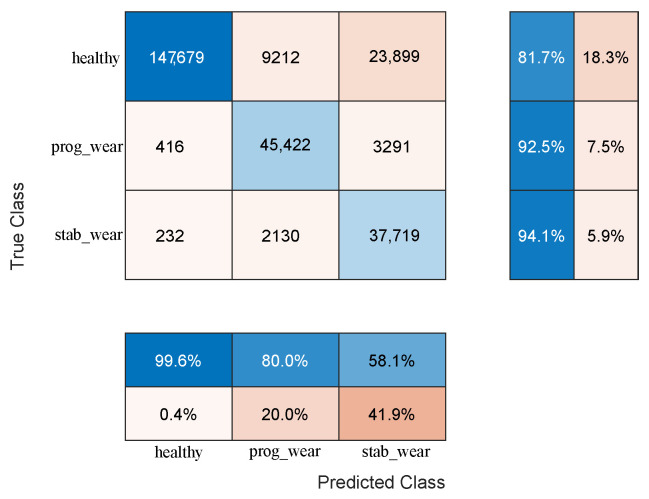
High-pass filtering—NHMC model.

**Figure A4:**
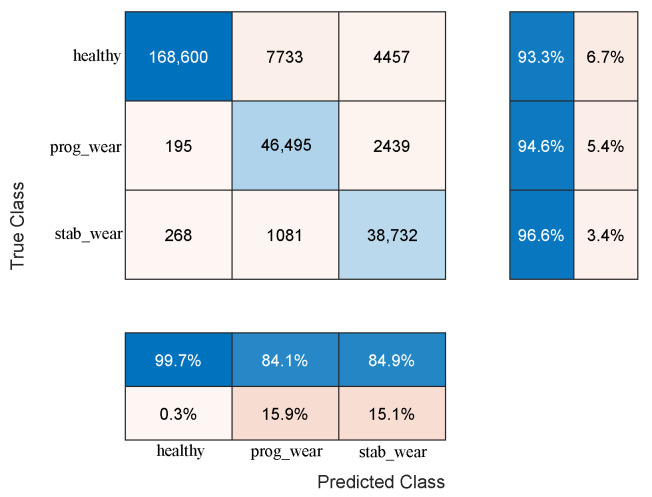
High-pass filtering—CHMC model.

**Figure A5:**
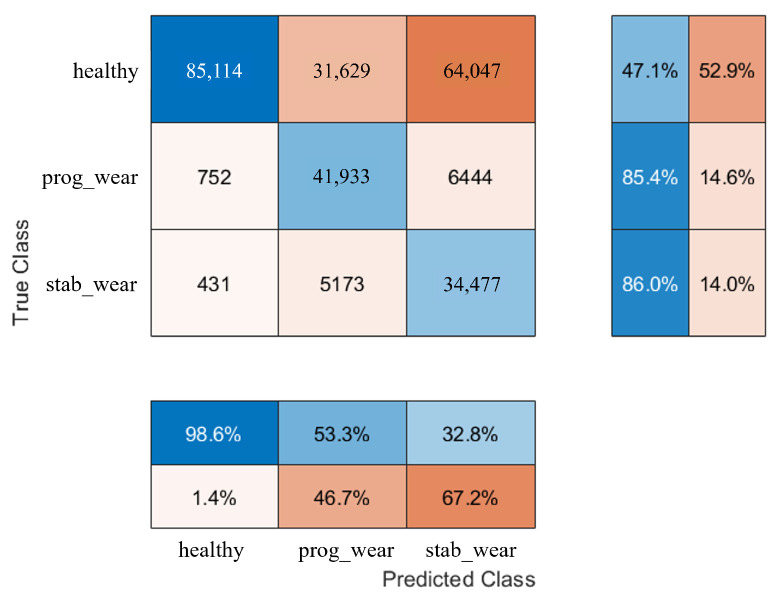
TFD—NHMC model.

**Figure A6:**
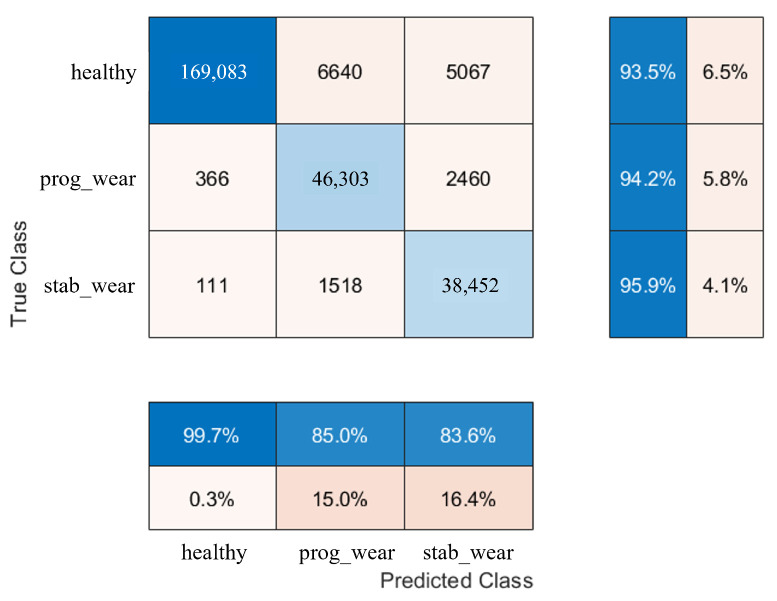
TFD—CHMC model.

**Figure A7:**
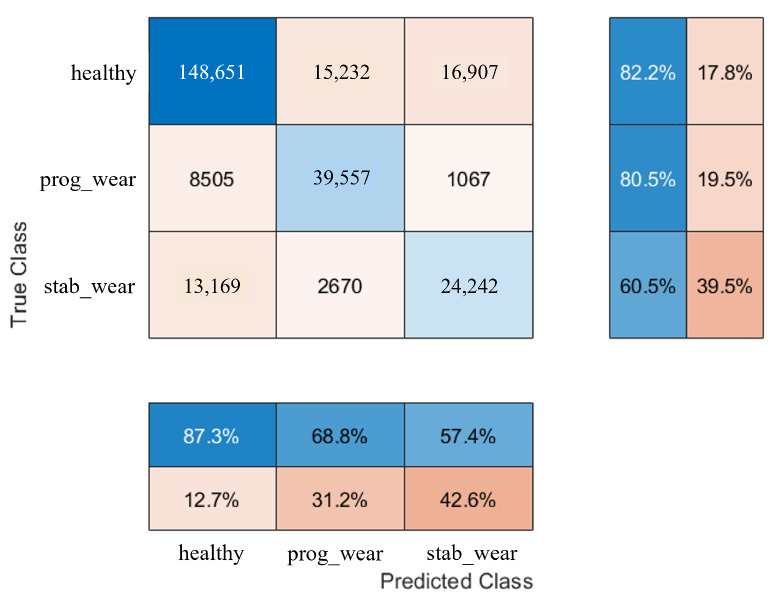
SVD—NHMC model.

**Figure A8:**
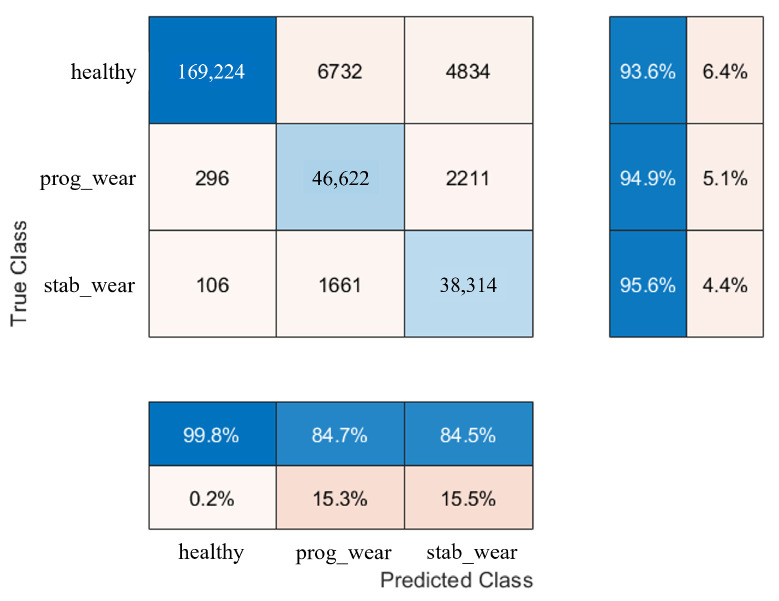
SVD—CHMC model.

**Figure A9:**
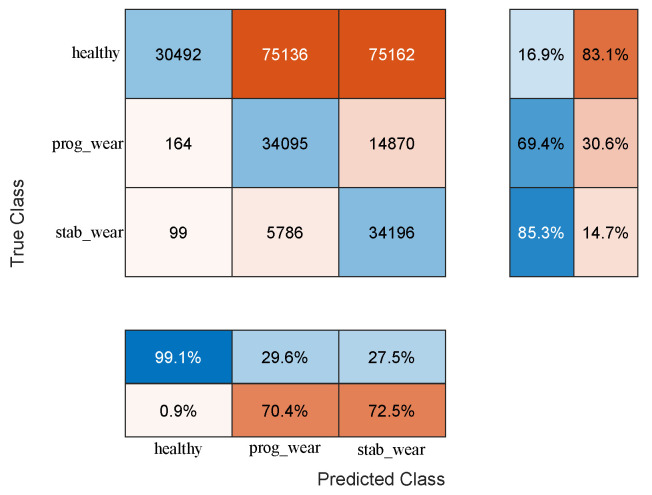
PCA + FFT—NHMC model.

**Figure A10:**
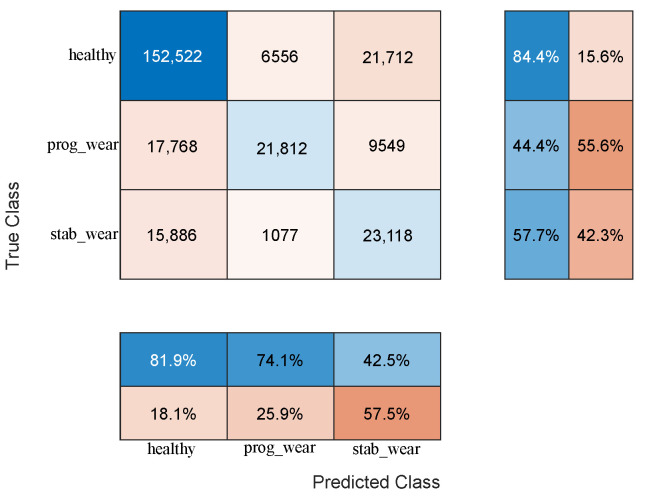
PCA + FFT—CHMC model.

This result seems to be coherent, since according to Figure 13, about 140 bases contribute significantly to the reconstruction of new signals. This method is therefore discarded for the remainder of our analysis. In conclusion, depending on the frequency range of the velocity profiles (predominantly high or low), a classical filtering technique may be sufficient for extracting a reduced base summarising the different employment profiles. Nevertheless, the SVD method is better at detecting motor failure than other methods. The second advantage is that it also provides the largest reduction. The performance of the model with unknown data projected onto a known database validates the extrapolation capabilities of the hybrid model. To support these comments, the $F_{1}-Score$ is calculated for each class (see Table A1). The results observed with the other methods in Table 5 demonstrate the ability of the model to adapt to new situations. This, in turn, has the effect of reducing the costs associated with modelling, as the model is not required to undergo a further learning process when a new situation arises. For each reduction method tested, details of the classification results are given in the appendices (Figure A1, Figure A2, Figure A3, Figure A4, Figure A5, Figure A6, Figure A7, Figure A8, Figure A9 and Figure A10).

Thanks to the integration of physical models in the fault diagnosis study, the CHMC model always manages to detect faults on extrapolated profiles better than the NHMC model, using reduction methods on input or non-input profiles (Table 5). The integration of physics makes it possible to distinguish more subtly whether the system is being used with new profiles or whether it is behaving abnormally.

In summary, dimensionality reduction is essential to overcome the challenge of limited model extrapolation, especially when models are implemented for specific operating conditions. In industrial equipment failure monitoring, it offers several advantages. Firstly, it reduces the complexity of the data by retaining essential information, allowing better generalisation of behaviour such as failure. Secondly, it eliminates noise by removing redundant or irrelevant dimensions, thereby highlighting critical features for monitoring. It also enables more efficient monitoring with fewer sensors, resulting in cost and resource savings. Finally, it improves analysts’ understanding of the underlying processes by simplifying the data while preserving their essence.

## 4. Results and Analysis

The study evaluates the performance of the CHMC model that integrates physical knowledge of an electric motor, compared to the NHMC model that does not use this information. Both models are evaluated on unbalanced data sets where the classes are distributed as 15.23% for “progressive failure”, 11.98% for “stable failure” and 72.79% for “healthy state”.

For usage profiles analogous to those in the training set, both models demonstrate comparable performance without reduction (see Table 2). However, the CHMC model exhibits superiority over the NHMC model in detecting true negatives (90% against 89%), as it is capable of leveraging the motor’s nominal physical behaviour to more effectively differentiate between the healthy and failure states (see Figure 3). The NHMC model encounters greater difficulty in distinguishing between the two failure states (83.92% against 86.56% for progressive failure and 89.14% against 89.41% for stabilized failure), resulting in less precise failure detection. Consequently, the CHMC model is more appropriate for predictive maintenance, where minimising false failure warnings is paramount.

When exposed to new usage profiles, the CHMC model adapts well, although it struggles slightly more than the NHMC model in detecting true negatives. The study tests the models on new input signals with a 42% increase in frequency range and a 2.5% increase in amplitude compared to the training data. The CHMC model still performs better in detecting true positives and handling new data, showing its greater extrapolation capability (see Figure 11 and Figure 12).

The employment of dimensionality reduction techniques, especially SVD, serves to reduce the complexity of the input data. As shown on Table 5, the CHMC model consistently outperforms the NHMC model when applied to reduced data, thereby demonstrating the efficacy of integrating physical models and dimensionality reduction in enhancing the model’s capacity to generalise, detect faults, and adapt to novel scenarios. This approach reduces the need for retraining, making the CHMC model a cost-effective and efficient solution for motor fault classification in industrial settings. In conclusion, the CHMC model, through the integration of physical knowledge and dimensionality reduction, provides a significant advantage in fault detection, model adaptation, and performance across varying usage profiles, ensuring more reliable and efficient predictive maintenance.

## 5. Conclusions and Future Works

### 5.1. Conclusions

In this study, a Cooperative Hybrid Model of Classification was explained in detail. The CHMC model offers a new approach to the challenges posed by conventional modelling when it comes to handling data from multiple system usage profiles at in a cost-effective manner. Such models require, first and foremost, access to real system data, as well as to part of the system’s physical models. Thus, one of the main results presented is the rapid construction of the hybrid model, provided that the system knowledge is accessible and/or implemented. This model has the advantage of being enriched over time, as new usage profiles appear.

As a first step, the newly implemented hybrid architecture was verified on the motor use case. The cooperative hybrid model slightly improves the classification of motor faults artificially injected into the simulated physical model. On known usage profiles, the performance improvement of the CHMC model is not significant compared with modelling without model hybridisation. However, no loss of performance is observed. The strength of the CHMC model lies above all in its ability to extrapolate when faced with new usage profiles. Indeed, the integration of the system’s physical models enables it to easily distinguish between the onset of abnormal behaviour, and, in particular, the onset of component failure. Since performance is preserved without the need to train again, even in the presence of new unknown data for the model, this could save time when monitoring a system in real time. The proposed approach based on dimensional reduction has shown that the model implemented is able to achieve equivalent performance by reducing the number of input data. This means that when a new usage profile is applied to the motor, the model seeks to break it down into the several profiles it has already encountered during its training period, in order to first identify and localize the fault. This type of modelling also provides a better understanding of the decisions made by the CHMC model. If the residuals do not oscillate around zero, the model will tend to indicate the onset of failure. In future work, we plan to implement a complementary algorithm to recognize whether high residuals reflect failure or a usage profile that is too far removed from the initial base of usage profiles. The CHMC model thus provides a starting point for measuring the explicability of predictive models.

In summary, the CHMC model provides an innovative approach to efficiently process data from multiple system usage profiles by facilitating the learning process and combining physical models of the system with real data. It is characterised by its ability to extrapolate to new scenarios, enabling effective monitoring and a better understanding of the model’s decisions.

### 5.2. Limitations and Future Works

For future endeavours, several limitations within the scope of this study warrant investigation. One of the main limitations relates to the methodology’s reliance on the fusion of existing physical models with a recurrent neural network (RNN), suggesting a presumption of familiarity or partial understanding of said physical models. Should this prerequisite not be met, the substitution of empirically derived knowledge from expert feedback on healthy behaviours may be warranted. Furthermore, the existing literature highlights the prevalence and efficacy of hybridising deep learning models, a prospect to be considered by integrating such hybridisation with the methodology established here.

Another limitation lies in the deliberate initial selection of reduction methods commonly used by physicists. An ongoing exploration involves juxtaposing the dimensionality reduction methods evaluated in this study with more advanced techniques such as kernel principal component analysis (k-PCA), linear discriminant analysis (LDA), t-distributed stochastic neighbour embedding (t-SNE), or locally linear embedding (LLE).

Although K-PCA and more complex structures, such as autoencoders, have been tested without significant improvements in model performance, this remains a promising avenue for future research, particularly for more complex systems with multiple interlocking subsystems.

In addition, while noise in the output data of physical models has been acknowledged, quantifying the resilience to noise and outliers remains a prospect for future investigations.

Finally, while the use case chosen in this study has potential applicability across different domains, careful analysis is essential to adapt the model to alternative systems. Indeed, while the overarching methodology aims for deployment across different systems, the direct transferability of expert physical knowledge (physical models, reduction base profiles) between systems is not guaranteed. Therefore, a comprehensive review is essential to ensure the adaptability and effectiveness of the model in different contexts.

As part of future work, the present methodology is to be evaluated through experimentation on authentic data from an alternative motor, characterised by divergent nominal properties when compared with a test bench and an additional modelled system.

## Figures and Tables

**Table 1:** Hyperparameters of the CHMC model.

Parameter	Setting
Learning rate	0.001
Epochs	80
Optimizer	Adam
Loss function	Weighted Cross Entropy

**Table 2:** Performance comparison between CHMC and NHMC.

Model	Training Loss	Accuracy	$F_{1}$-Score
CHMC model	0.061	0.97	0.90
NHMC model	0.068	0.97	0.89

**Table 3:** $F_{1}-Score$ for each class on known profiles.

Model	Healthy	Progressive Failure	Stabilized Failure
CHMC model	96.95	86.56	89.41
NHMC model	96.06	83.92	89.14

**Table 4:** $F_{1}-Score$ of the CHMC model at different noise levels.

Noise Level	20	15	10
CHMC model	90.11	88.64	85.72

**Table 5:** Comparison of reduction methods between the CHMC model and the NHMC model.

Reduction Method	Model	Accuracy (%)	$F_{1}$-Score (%)	Number of Profiles in the Database
None	CHMC	84.74	69.08	270
	NHMC	86.75	61.74	
Low frequencies	CHMC	97.50	91.76	243
	NHMC	83.78	58.95	
High frequencies	CHMC	97.50	91.93	28
	NHMC	94.10	82.46	
TFD	CHMC	97.50	91.73	172
	NHMC	83.78	58.95	
SVD	CHMC	97.56	91.93	20
	NHMC	86.59	72.60	
PCA + FFT	CHMC	81.74	62.53	20
	NHMC	74.64	37.33	

## Data Availability

The raw data supporting the conclusions of this article will be made available by the authors on request.

## Nomenclature

ANN	Artificial Neural Network
BB	Black Box
BiLSTM	Bi-directional Long Short-Term Memory
CHMC	Cooperative Hybrid Model of Classification
CNN	Convolutional Neural Network
DC	Direct-Current
DFT	Discrete Fourier Transform
DL	Deep Learning
FFT	Fast Fourier Transform
HDL	Hybrid Deep Learning
HM	Hybrid Model
HPDM	Hybrid Physics-based Data-driven Models
k-PCA	Kernel Principal Component Analysis
LDA	Linear Discriminant Analysis
LLE	Locally Linear Embedding
LSTM	Long Short-Term Memory
NHMC	Non-Hybrid Model of Classification
NN-APM	Neural networked Augmented Physics Models
PDE	Partial Differential Equations
PINN	Physics-Informed Neural Networks
RNN	Recurrent Neural Network
SNR	Signal-to-Noise Ratio
SVD	Singular Value Decomposition
t-SNE	t-Distributed Stochastic Neighbour Embedding
WB	White Box

## Appendix A

**Table A1:** $F_{1}-Score$ for each class on unknown profiles.

Model	Healthy	Progressive Failure	Stabilized Failure
NHMC	76.85	60.26	52.72
CHMC	85.78	61.09	59.38
